# Single-cell RNA sequencing reveals molecular and functional platelet bias of aged haematopoietic stem cells

**DOI:** 10.1038/ncomms11075

**Published:** 2016-03-24

**Authors:** Amit Grover, Alejandra Sanjuan-Pla, Supat Thongjuea, Joana Carrelha, Alice Giustacchini, Adriana Gambardella, Iain Macaulay, Elena Mancini, Tiago C. Luis, Adam Mead, Sten Eirik W. Jacobsen, Claus Nerlov

**Affiliations:** 1 MRC Molecular Hematology Unit, Weatherall Institute of Molecular Medicine, John Radcliffe Hospital, University of Oxford, Oxford OX3 9DS, UK; 2 Institute for Stem Cell Research, University of Edinburgh, Edinburgh EH16 4UU, UK; 3 Haemopoietic Stem Cell Laboratory, Weatherall Institute for Molecular Medicine, University of Oxford, Oxford OX3 9DS, UK; 4 EMBL Mouse Biology Program, 00015 Monterotondo, Italy

## Abstract

Aged haematopoietic stem cells (HSCs) generate more myeloid cells and fewer lymphoid cells compared with young HSCs, contributing to decreased adaptive immunity in aged individuals. However, it is not known how intrinsic changes to HSCs and shifts in the balance between biased HSC subsets each contribute to the altered lineage output. Here, by analysing HSC transcriptomes and HSC function at the single-cell level, we identify increased molecular platelet priming and functional platelet bias as the predominant age-dependent change to HSCs, including a significant increase in a previously unrecognized class of HSCs that exclusively produce platelets. Depletion of HSC platelet programming through loss of the FOG-1 transcription factor is accompanied by increased lymphoid output. Therefore, increased platelet bias may contribute to the age-associated decrease in lymphopoiesis.

## Abstract (web summary)

With age, haematopoietic stem cells (HSCs) produce more myeloid than lymphoid cells, affecting adaptive immunity. By combining HSC single cell transcriptomics with functional studies, Grover *et al*. find that platelet production is also increased in old murine HSCs and show that the FOG-1 transcription factor contributes to the age-dependent platelet bias.

## Graphical abstract

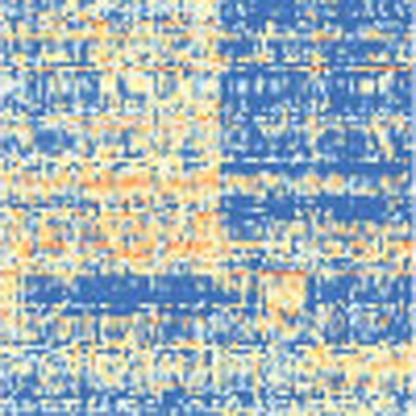

Changes to the properties of tissue stem cell populations underlie physiological alterations and diminished regenerative potential associated with mammalian ageing^1^. One of the key age-related changes to haematopoiesis is a decrease in the production of erythrocytes and lymphoid cells (B- and T-cells), contributing to age-associated anaemia and a progressive decline in adaptive immunity^2^,^3^,^4^. Intrinsically altered function of haematopoietic stem cells (HSCs) contributes significantly to these changes, as the increased ratio of myeloid-to-lymphoid output is conserved on transplantation of aged mouse HSCs into young recipients^5^, a finding replicated with human HSCs (ref. 6). Single-cell transplantations have established that the HSC compartment is functionally heterogeneous, with stably myeloid- and lymphoid-biased HSC subsets existing already in young mice^7^,^8^,^9^, and that myeloid-biased HSCs become dominant with age^10^,^11^, leading to the proposal that age-related myeloid lineage bias is due to superior self-renewal of myeloid-biased compared with lymphoid-biased HSCs. While technical limitations precluded the assessment of platelet output of transplanted HSCs in previous studies, we recently used a *Vwf-EGFP* transgene to measure platelet output from single HSCs of young adult mice, establishing that myeloid-biased HSCs also typically produce high levels of platelets, and that a subset of HSCs exist with a distinct and stable platelet bias^12^. The cellular complexity of the HSC compartment is therefore greater than previously appreciated, and an understanding of how the lineage-bias of HSCs changes on ageing will require investigation of the prevalence and function of all identified HSC subtypes in aged mice and humans.

In addition to age-dependent changes in the lineage output of the HSC compartment, there is also evidence supporting that other intrinsic properties of HSCs are altered with age. Aged HSCs have been suggested to engraft with a lower frequency than young HSCs, and at the single-cell level contribute less to peripheral blood reconstitution^5^,^11^,^13^,^14^. Moreover, comparison of the gene expression profiles of young and old mouse HSC cell populations has identified a number of processes and pathways upregulated in aged HSCs, including NF-κB pathway activation, DNA repair and chromatin remodelling^13^. In addition, an increase in myeloid lineage-associated and concomitant decrease in lymphoid lineage-associated gene expression has been observed^6^,^15^, and more recently also an increase in platelet gene expression^16^. Finally, upregulation of Wnt5a in aged HSCs and associated Cdc42-mediated loss of polarity^17^,^18^ have been implicated in myeloid bias and loss of reconstitution capacity, potentially linking intrinsic changes to HSCs to altered lineage output.

While some aspects of HSC ageing, such as lineage output and reconstitution capacity, have been assessed at the single-cell level, the associated gene expression changes have not. Critically, bulk cell population-based analysis of HSC gene expression cannot determine if observed alterations associated with aging occur homogeneously throughout the HSC compartment, or in a subset of HSCs. Consequently, the molecular mechanisms underlying HSC ageing remain poorly understood.

To identify age-dependent intrinsic molecular changes to HSCs, we have therefore taken advantage of recent progress in single-cell transcriptomics to systematically compare individual HSC transcriptomes from young and old mice, and combined this analysis with functional studies of single HSC that include readout of their platelet production. We find that HSC ageing is accompanied by a coordinated upregulation of platelet-lineage gene expression, both in terms of the number of platelet-specific genes expressed per HSC and of their expression level. This is mirrored by a 50-fold increase in the abundance of platelet-primed HSCs as defined by *Vwf-EGFP* expression^12^, and by functional platelet bias at the single-cell level. Most notably, we observe that a very high proportion of aged HSCs almost exclusively produce platelets, and that when accounting for these previously unrecognized platelet-restricted HSCs, there is in fact no age-dependent decrease in the frequency of HSC capable of engraftment on transplantation. Aged HSCs are therefore not impaired in their engraftment, but rather become highly platelet-biased with age. Moreover, depleting the platelet programming from HSCs through genetic ablation of the FOG-1 transcription factor is accompanied by an increase in lymphoid lineage output, suggesting that the platelet gene expression programme may contribute to the suppression of lymphoid output that is a key characteristic of aged HSCs.

## Results

### Gene expression profiling of young and old HSCs

**Figure 1:**
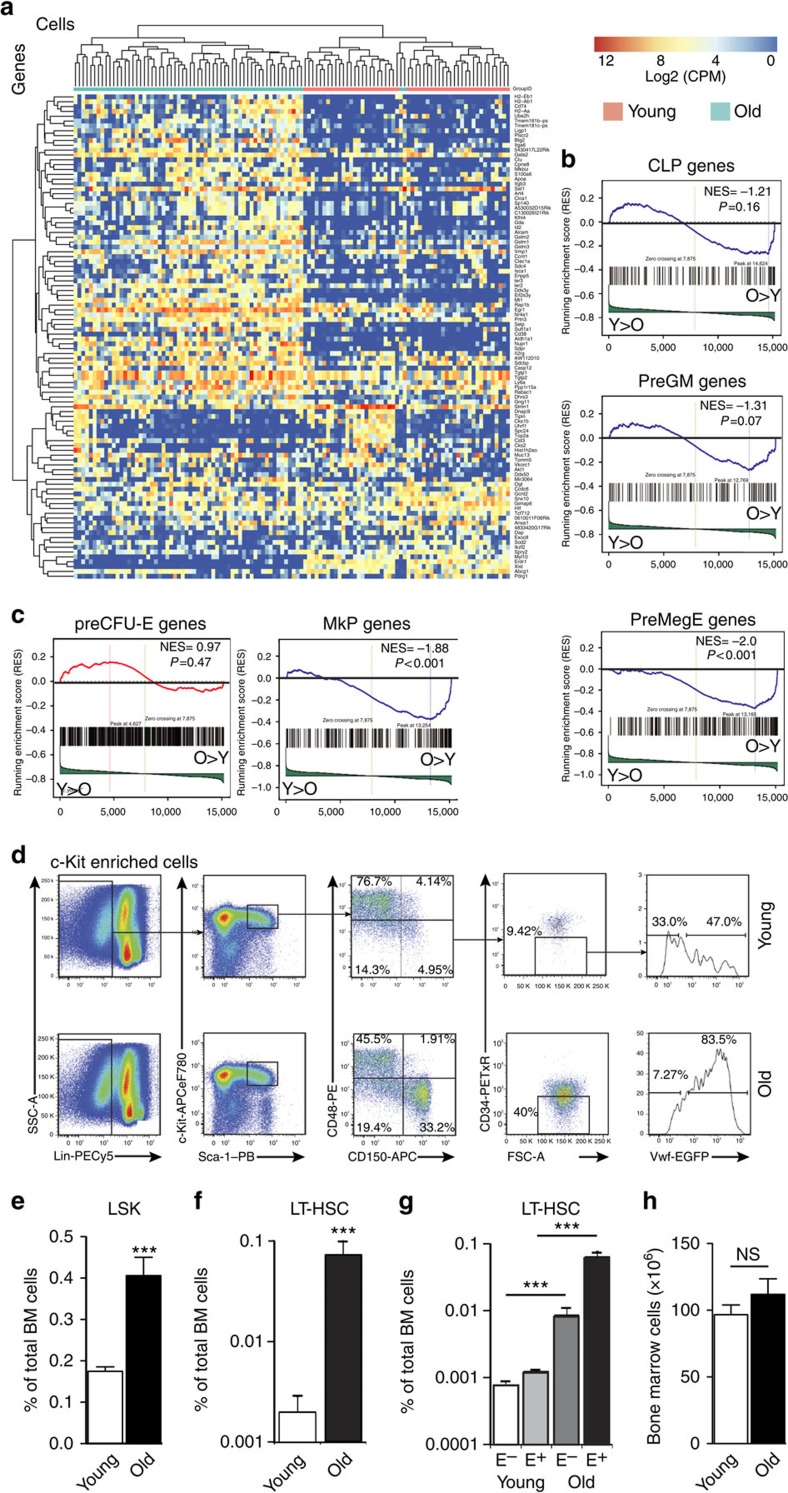
Expansion of platelet-primed HSCs in old mice. (**a**) Hierarchical clustering of young and old LSKCD150^+^CD48^−^ HSC single-cell transcriptomes using the top 100 variable genes from components 1–3 of combined PCA of young and old HSCs. Data are from two independent experiments (young mice *N*=1; old mice *N*=1). (**b**) Signature enrichment plots from GSEA analysis using CLP, preGM and preMegE signature gene sets. The values indicated on individual plots are the Normalised Enrichment Score (NES) and *P*-value of the enrichment (permutation analysis). Note the significant upregulation of preGM, and preMegE signatures in old HSCs. (**c**) Signature enrichment as in **b** using CFU-E and MkP signature gene sets. Note the significant upregulation of the MkP signature in old HSCs. (**d**) Representative FACS plots for isolation of Vwf^-^ and Vwf^+^ LT-HSCs (defined as LSKCD48^−^CD150^+^CD34^−^ cells) in BM from young and old *Vwf-EGFP* transgenic mice after performing c-Kit enrichment. Numbers indicate percentage of the parental gate. (**e**) Frequencies of LSK cells in total BM in young (*N*=24) and old (*N*=5) mice. Data are from five independent experiments. Values show mean±s.e.m. ***P<0.001. (Student's *t*-test). (**f**) Frequencies of LSKCD48^−^CD150^+^CD34^−^ LT-HSCs as percentage of live singlets in young (*N*=31) and old (*N*=7) mice. Note: Log_10_ scale. Data are from six independent experiments. (**g**) Frequencies of Vwf^-^ and Vwf^+^ LT-HSCs as percentage of live singlets in young (*N*=31) and old (*N*=7) mice. E^−^, Vwf-EGFP^−^; E^+^, Vwf-EGFP^+^. Note: Log_10_ scale. Data are from six independent experiments. (**h**) Bone marrow cellularity of young (*N*=29) and old (*N*=7) *Vwf-EGFP* transgenic mice. Cellularity was determined from two iliac cristae, two tibiae and two femora after RBC lysis. Values show mean±s.e.m. ***P<0.001. (Student's *t*-test); NS, not significant. Data are from six independent experiments.

To establish the intrinsic molecular changes to HSCs occurring with ageing, we performed single-cell RNA sequencing of young (2–3 months) and old (20–25 months) HSCs, stringently defined as Lin^−^Sca-1^+^c-Kit^+^1 (LSK) CD150^+^CD48^−^ (ref. 19). A total of 61 young and 74-old single HSC transcriptomes were generated, using the C1 Auto-prep system and Illumina-based deep sequencing, 52 and 62 of which, respectively, were used for analysis following quality control, as described in ref. 20. Principal component analysis (PCA)-based clustering was performed of these cells using genes with coefficient of variation >1 and average expression >1 count per million (CPM) across the whole data set. We identified the 100 genes with the highest absolute correlation coefficient within the PCA component loadings of the first 3 PCA components. These genes showed high expression and significant variation (Supplementary Fig. 1). Unsupervised clustering of the transcriptomes using this gene set showed that young and old HSCs formed separate clusters (Fig. 1a), showing that intrinsic changes occurring to the vast majority of HSCs during ageing significantly exceed the differences existing between individual HSCs in young and old mice. The principal pathways enriched in young HSCs were involved in cell cycle progression (Supplementary Data 1). This was due to an increased frequency of expression of cell cycle regulators (including *Ccna2*, *Ccnb1*, *Cdk1* and *Cdc25c*; Supplementary Fig. 2a), consistent with the reported higher frequency of cell division of young HSCs (refs 21, 22). In contrast, pathways involved in the immediate-early response to growth factor signalling were enriched in old HSCs (Supplementary Data 2), correlating with a greater proportion of cells expressing immediate-early genes (*Egr1*, *Egr3*, *Ier3* and *Junb*; Supplementary Fig. 2b). Notably, among the top 60 genes upregulated in old HSCs (Supplementary Data 3) 6 were associated with platelet-lineage differentiation (*Clu*, *Selp*, *Sdpr*, *Itgb3*, *Fhl1* and *Tgm2*), all of which were also expressed more frequently in old HSCs (Supplementary Fig. 2c). The latter observation indicated that age-dependent intrinsic differences between young and old mice indeed have the potential to influence lineage output already at the HSC level. To systematically identify the lineage programs altered during HSC ageing, we performed Gene Set Enrichment Analysis (GSEA)^23^ using previously defined gene sets^24^ specific for common lymphoid progenitors (CLPs), bi-potent granulocyte/macrophage progenitors (preGM) and megakaryocyte/erythroid progenitors (preMegEs)^25^, representing the three major lineage branches downstream of HSCs. This analysis showed a strong enrichment of preMegE-specific genes (NES=2; *P*<0.001, permutation analysis) in old HSCs, with a lesser enrichment of preGM-specific genes (NES=1.31; *P*=0.07, permutation analysis) and no significant change to CLP-specific gene expression (Fig. 1b). Similar analysis using gene sets representing committed megakaryocyte progenitors (MkPs) and erythroid progenitors (preCFU-Es) showed a selective enrichment of MkP-specific genes (NES=1.88; *P*<0.001, permutation analysis) in old HSCs (Fig. 1c).

These results confirmed a significant and selective age-dependent increase in HSC Mk/platelet-lineage programming. To investigate the cellular basis for this increase, we analysed aged mice carrying the *Vwf-EGFP* transgene previously shown to identify molecularly platelet-primed and functionally platelet-biased HSCs in young mice^12^. As expected from previous studies^5^,^26^,^27^ both the HSC-containing LSK population and highly purified HSCs (defined as LSKCD150^+^CD48^−^CD34^−^ (refs 19, 28) were significantly expanded (Fig. 1d–f). We observed that this increase in HSC numbers was principally driven by an increase in *Vwf-EGFP*^+^ (henceforth Vwf^+^) LT-HSCs, which showed a far greater expansion (52 fold) than Vwf^−^ HSCs (11 fold) in old mice (*P*<10^−5^; Student's *t*-test) (Fig. 1g), demonstrating that the observed age-dependent increase in molecular platelet bias correlated with a selective expansion of platelet-primed Vwf^+^ HSCs. No change to the total bone marrow (BM) cellularity was observed in aged mice (Fig. 1h).

### Platelet-biased HSCs preferentially expand during ageing

**Figure 2:**
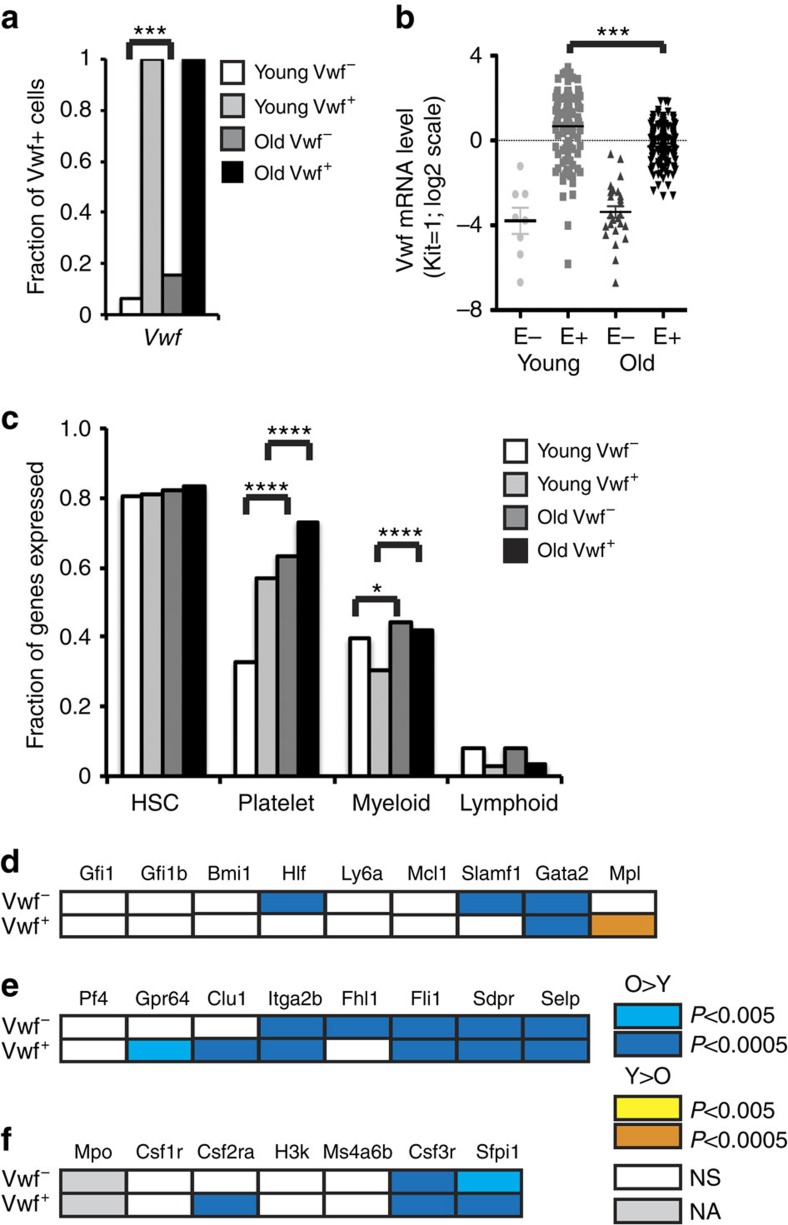
Aged LT-HSCs show increased transcriptional priming of platelet- and myeloid-lineage genes. (**a**) Bars show the frequency of cells in which *Vwf* expression was detected within the indicated LT-HSC populations by single-cell PCR. Number of cells analysed: young Vwf^−^, 123; old Vwf^−^, 167; young Vwf^+^, 111; old Vwf^+^, 163. ***P<0.001 (Student's *t*-test). Data are from five independent experiments (young mice *N*=3, old mice *N*=2) and a total of 16 BioMark 48.48 Dynamic Arrays. (**b**) *Vwf* expression level in single LT-HSCs from **a**. Dots represent individual expressing cells. Bars indicate the average expression level for positive cells in each population. E–, *Vwf-EGFP*^−^; E+, *Vwf-EGFP*^+^. ***P<0.001 (Student's *t*-test). (**c**) Bars show the frequency of expression of genes associated with HSC function and platelet, myeloid and lymphoid differentiation in single HSCs from **a**. The frequencies of co-expression were compared between old and young Vwf^−^ LT-HSCs (O– versus Y–), and between old and young Vwf^+^ LT-HSCs (O+ versus Y+) using the Kolmogorov–Smirnov test, and the level of significance is indicated. Data on individual genes are shown in Supplementary Fig. 2. (**d**) Diagram showing changes during ageing to the average expression level of the indicated HSC-associated genes in single Vwf^+^ and Vwf^−^ LT-HSCs from **a**. Detailed data in Supplementary Fig. 3. (**e**) Diagram showing changes during ageing to the average expression level of the indicated platelet-lineage-specific genes in single Vwf^+^ and Vwf^−^ LT-HSCs from **a**. Detailed data in Supplementary Fig. 4. (**f**) Diagram showing changes during ageing to the average expression level of the indicated myeloid lineage-specific genes in single Vwf^+^ and Vwf^−^ LT-HSCs from **a**. Data from Supplementary Fig. 5.

To determine if the age-dependent increase in HSC platelet gene expression was a general property of the HSC compartment, or due to the selective expansion of the Vwf^+^ HSC subset, we specifically investigated the age-dependent changes to HSC lineage priming of Vwf^+^ and Vwf^−^ HSCs at the single-cell level using targeted micro-fluidics-based quantitative PCR. Both the frequency of expression and the expression level of genes affiliated with the Mk/platelet, myeloid and lymphoid lineages, as well as genes associated with HSC function, were assessed in single Vwf^+^ and Vwf^−^ HSCs. We first validated the approach by measuring *Vwf* expression in the sorted HSC populations. *Vwf* expression was uniformly detected in Vwf^+^ HSCs (both young and old), but only at low levels in a small fraction of Vwf^−^ LT-HSCs (Fig. 2a,b), showing that messenger RNA expression can be detected with high efficiency using this methodology, and that different levels of expression can be accurately discriminated. Importantly, and as expected, HSC-associated genes were highly and uniformly expressed in all HSC populations (Fig. 2c,d; Supplementary Figs 3a and 4). For platelet-lineage gene expression, we observed a highly significant, age-dependent increase in the frequency of expression, not only in Vwf^+^ HSCs but also in Vwf^−^ HSCs (Fig. 2c,e; Supplementary Fig. 3b), with the majority of platelet-lineage genes being more highly expressed in Vwf^+^ and Vwf^−^-aged HSCs (Supplementary Fig. 5). In contrast, an age-dependent increase in the frequency of myeloid priming was highly significant only in Vwf^+^ HSCs (Fig. 2c, Supplementary Fig. 3c), with the expression level of a subset of myeloid genes showing upregulation (Fig. 2f; Supplementary Fig. 6). No change to the frequency of HSCs with lymphoid gene expression was observed in either Vwf^+^ or Vwf^−^ HSCs (Fig. 2c; Supplementary Fig. 3d). Together, these results demonstrate that during ageing platelet-lineage priming of HSC increases at three different levels: through an increased proportion of Vwf^+^ platelet-primed HSCs (ref. 12), an increase in the frequency of platelet-specific gene expression in both Vwf^−^ and Vwf^+^ HSCs, and an increase in the level of expression of platelet-lineage genes in individual HSCs. In addition, in the Vwf^+^ HSCs, representing the most highly platelet-primed HSCs, we observed a parallel increase in myeloid gene expression. Importantly, these changes occur in single cells that homogeneously co-express multiple HSC-specific genes, indicating that functional HSCs become increasingly platelet-primed with age.

### Aged HSCs are functionally platelet biased

**Figure 3:**
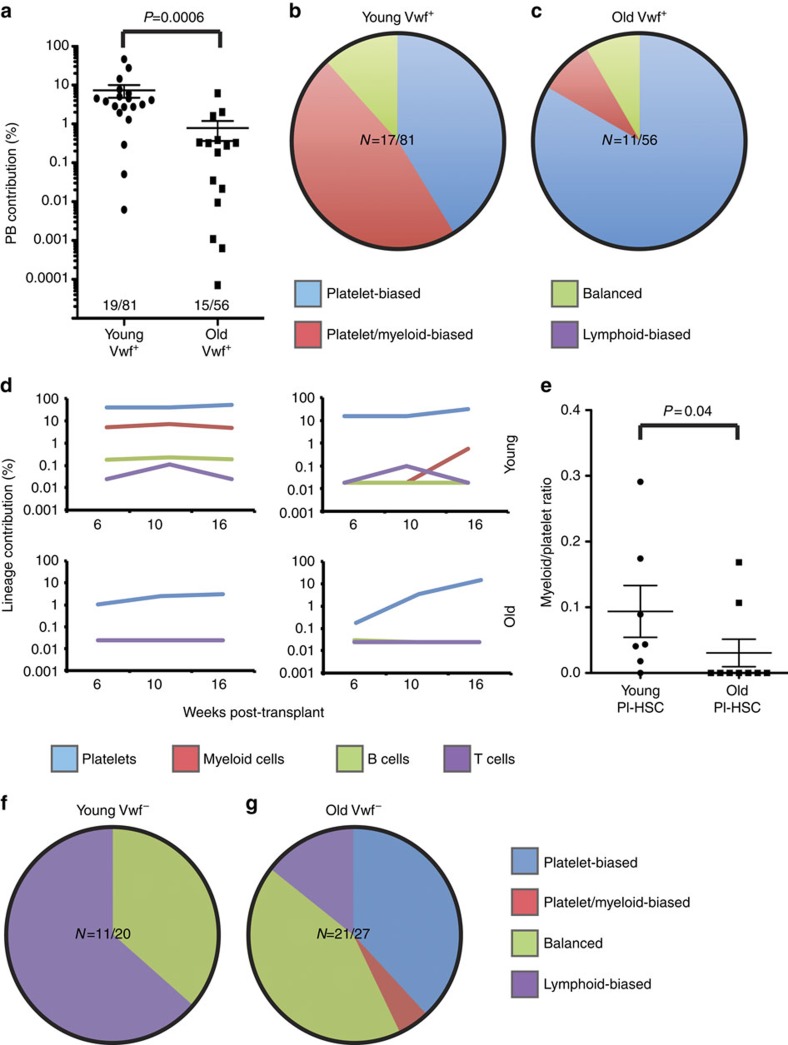
Aged HSCs show increased platelet bias at the single-cell level. (**a**) Contribution of transplanted single young and old Vwf^+^ LT-HSCs to peripheral blood reconstitution, expressed as the average of their contributions to peripheral blood platelets, myeloid cells, B cells and T cells. The frequency of reconstitution is indicated as the ratio between reconstituted and transplanted mice. The significance of the difference in reconstitution between young and old Vwf^+^ LT-HSCs is shown (Mann–Whitney *U*-test). (**b**) Distribution of HSC subtypes in young Vwf^+^ LT-HSCs from **a**, excluding those with no contribution of at least 0.1% to at least one lineage. The number of clones fulfilling this criterion is indicated. (**c**) Analysis as in **b** for old Vwf^+^ LT-HSCs. (**d**) Representative examples of peripheral blood reconstitution by Pl-HSCs from young (top) and old (bottom) Vwf^+^ LT-HSC. (**e**) The ratio of myeloid to platelet reconstitution for Pl-HSCs derived from young and old Vwf^+^ LT-HSCs, respectively. The frequency of clones with only detectable platelet output was significantly higher for old Vwf^+^ LT-HSCs (Fisher's exact test). (**f**) Distribution of HSC lineage outputs in mice transplanted with five young Vwf^−^ LT-HSCs, excluding those with contribution <0.1% to all lineages. The number of clones fulfilling this criterion is indicated. (**g**) Analysis as in **f** for old Vwf^−^ LT-HSCs. Number of recipient mice transplanted, young Vwf^+^ *N*=81; young Vwf^−^ *N*=20; old Vwf^+^=56, old Vwf^−:^ *N*=27. Time points for peripheral blood analysis post-transplantation are indicated in plots. Data are from five independent experiments (young *N*=3, old *N*=2).

To investigate the functional changes accompanying the observed age-dependent molecular changes to HSC lineage priming, we transplanted single or limiting numbers of Vwf^+^ and Vwf^−^ HSCs to investigate clonally reconstituted mice, specifically single HSC contributions to peripheral blood myeloid cells (defined as Mac-1^+^), T cells (CD4^+^ or CD8^+^), B cells (CD19^+^) and platelets (CD150^+^), in the latter case using enhanced green fluorescent protein (EGFP) expressed by the *Vwf-EGFP* transgene to identify the single-HSC platelet contribution^12^. By transplanting single young and old CD45.2 LT-HSCs, along with CD45.1 competitor BM, into young CD45.1 recipient mice, we observed that Vwf^+^ HSCs from young and old mice gave rise to HSC clones with similar frequencies (Young Vwf^+^ HSCs: 23%; old Vwf^+^ HSCs: 27%; *P*=0.69, Fisher's exact test; Fig. 3a). However, the average peripheral blood contribution of old HSCs was significantly lower than that of young HSCs (Fig. 3a), confirming an age-dependent intrinsic decrease in the ability of HSCs to produce mature blood cells. We previously described that young HSCs show at least four distinct types of lineage bias in single-cell transplantation assays^12^, namely platelet bias (Pl), platelet/myeloid bias (Pl/My), lymphoid/myeloid balanced output (Ba) and lymphoid bias (Ly). In mice transplanted with single young Vwf^+^ HSCs, we observed that Pl/My-HSCs and Pl-HSC dominated, with no Ly-HSCs observed (Fig. 3b), as previously described in ref. 12 defining lineage bias as a threefold higher lineage output compared with the remaining lineages, measured 10 and 16 weeks post-transplant. The analysis of clonal bias was restricted to clones showing >0.1% contribution to at least one lineage, to allow reliable detection of all cell lineages within each clone. When single cells from the same Vwf^+^ HSC population isolated from BM of old mice were transplanted, we observed a significant increase in the proportion of platelet-biased clones, in that >80% of Vwf^+^ HSC-derived clones were platelet biased (*P*=0.02; Fisher's exact test), again with no Ly-HSCs detected (Fig. 3c). Furthermore, we observed that the platelet-biased clones derived from aged HSCs were highly platelet-restricted in their reconstitution potential, as in most cases only platelet output was detected, whereas platelet-biased HSCs derived from young mice also typically generated myeloid cells (Fig. 3d,e; *P*=0.04; Fisher's exact test). We transplanted young Vwf^−^ HSCs at 5 cells per mouse, since their frequency of engraftment was found to be low when transplanted as single cells^12^. Young Vwf^−^ HSCs generated, as expected^12^, clones with high lymphoid output (Ba-HSCs, Ly-HSCs), and no Pl- or Pl/My-HSCs (Fig. 3f). In contrast, aged Vwf^−^ HSCs generated fewer lymphoid-biased clones, with a corresponding increase in Pl- and Pl/My-HSCs (Fig. 3g), again consistent with their increased molecular platelet priming. Both the increased proportion of Pl-HSCs (*P*=0.03; Fisher's exact test) and the decrease in Ly-HSCs (*P*=0.012; Fisher's exact test) were significant. Based on the frequency of reconstituted mice, the frequency of repopulation by Vwf^−^ HSCs was estimated as 16% in young mice and 30% in old mice using Poisson distribution of independent events. Therefore, also for Vwf^−^ LT-HSCs, there was no decrease in the repopulation frequency of aged HSCs when platelet-biased HSCs were also included in the reconstitution measurement.

**Figure 4:**
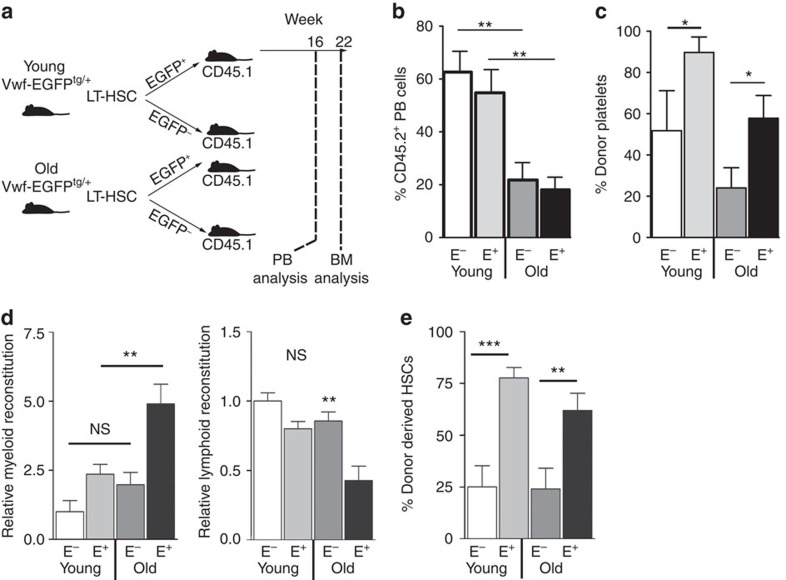
Aged Vwf^+^ LT-HSCs show increased intrinsic myeloid bias at the population level. (**a**) Experimental design to compare *in vivo* lineage output of transplanted young and old Vwf^+^ and Vwf^−^ LT-HSCs. (**b**) Donor contribution of young and old Vwf^−^ and Vwf^+^ LT-HSCs to total peripheral blood leucocytes at 16 week post-transplantation. Young Vwf^−^: *N*=5; young Vwf^+^: *N*=11; old Vwf^−^: *N*=9, old Vwf^+^: *N*=12). E^−^: Vwf-EGFP^−^; E^+^: Vwf-EGFP^+^. Data are from four independent competitive transplantation experiments. (**c**) Donor contribution to peripheral blood platelets measured at 16 week post-transplantation in experiments described in **b**. (**d**) Relative contribution of test cells to output of myeloid (left panel) and lymphoid (right panel) cells at 16 weeks post-transplantation from experiment described in **b**. Values were normalized to the level of CD45.2 chimerism (**b**) and are expressed relative to young Vwf^−^ recipients (=1). (**e**) Test cell contribution to BM LSKCD150^+^CD48^−^CD34^−^ LT-HSCs in mice from **b** 22 weeks after transplantation. E^−^, Vwf-EGFP^−^; E^+^, Vwf-EGFP^+^. Data are from three independent experiments. All data are mean values±s.e.m. *P<0.05; **P<0.01; ***P<0.001 (Student's *t*-test).

To determine the overall effect of the age-dependent changes to HSC subtype prevalence on lineage output, we transplanted bulk populations of Vwf^+^ and Vwf^−^ HSCs (CD45.2) from young and old mice into lethally irradiated recipients (CD45.1) along with wild-type (young) competitor BM (CD45.1; Fig. 4a). Old Vwf^+^ and Vwf^−^ HSCs both showed 2–3 fold lower long-term reconstitution of peripheral blood leucocytes (Fig. 4b), and both young and old Vwf^+^ HSCs showed elevated long-term platelet output compared with their Vwf^−^ counterparts (Fig. 4c). Notably, the platelet contribution from old Vwf^+^ HSCs exceeded the leucocyte output by threefold, whereas from young HSCs this difference was less pronounced (1.5 fold), consistent with the increased platelet bias observed at the single HSC level. After normalization to the total leucocyte output (to allow direct comparison between young and old HSCs) it became evident that old Vwf^+^ HSCs showed higher myeloid and lower lymphoid long-term reconstitution than young Vwf^+^ HSCs, with a similar, but non-significant, trend observed for Vwf^−^ HSCs (Fig. 4d). The increased myeloid-to-lymphoid output ratio paralleled the increased molecular platelet and myeloid priming observed at the single-cell level, which was significant when comparing young and old Vwf^+^ LT-HSCs, but marginal when comparing young and old Vwf^−^ LT-HSCs. Old and young HSCs showed similar reconstitution of the HSC compartment 5 months post-transplantation. Interestingly, however, Vwf^+^ HSCs in both cases replenished the HSC compartment more efficiently than Vwf^−^ HSCs (Fig. 4e). It has previously been reported that old HSCs show higher ability than young HSCs to reconstitute the HSC compartment, on a per cell basis, after transplantation^15^. Our results show that this may reflect the higher proportion of Vwf^+^ HSCs (which reconstituted the HSC compartment more efficiently than Vwf^−^ HSCs) in old mice. From these results we conclude that both the proportion of Vwf^+^ HSCs and their intrinsic platelet and myeloid bias increase during ageing.

### Progenitor platelet bias in aged haematopoiesis

**Figure 5:**
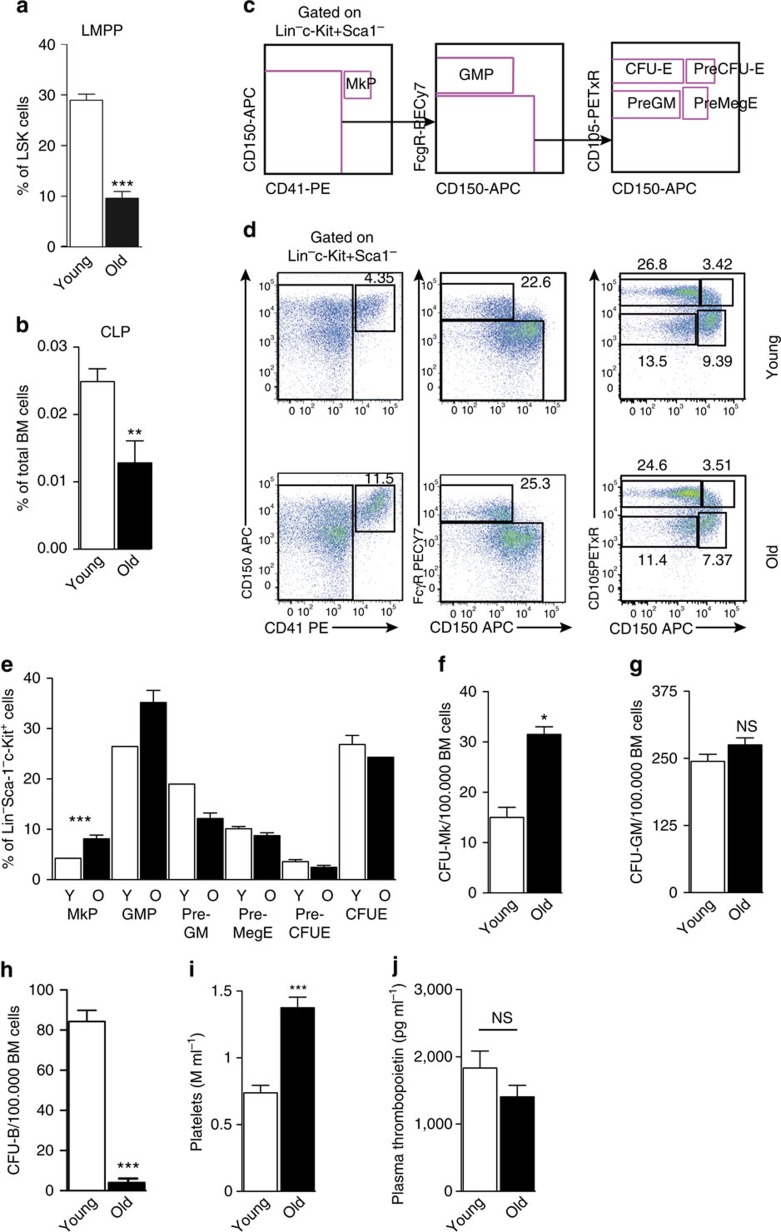
Alterations to phenotypic and functional progenitor levels in old mice. (**a**) Lymphoid-primed multi-potent progenitors (LMPPs) as percentage of LSK cells in young (*N*=15) and old (*N*=7) mice. Data are from five independent experiments. (**b**) Frequencies of CLPs in total BM in young (*N*=9) and old (*N*=6) mice. Data are from five independent experiments. (**c**) Gating strategy for identification of myelo-erythroid progenitor populations. (**d**) Representative FACS plots of myelo-erythroid progenitors in BM from young (top) and old (bottom) *Vwf-EGFP* mice. Numbers indicate percentage of Lin^−^Sca-1^−^c-Kit^+^ cells. (**e**) Frequencies of myelo-erythroid progenitor cells as percentage of Lin^−^Sca-1^−^c-Kit^+^ cells in young (Y: *N*=6) and old (O: *N*=6) mice. Data are from six independent experiments. (**f**) Frequencies of CFU-Mk in unfractionated BM from young (*N*=3) and old (*N*=2) mice. Data are from a single experiment. (**g**) Frequencies of CFU-GM in unfractionated BM from young (*N*=3) and old (*N*=2) mice. Data are from a single experiment. (**h**) Frequencies of CFU-B in unfractionated BM from young (*N*=3) and old (*N*=2) mice. Data are from a single experiment. (**i**) Platelet counts in peripheral blood from young (*N*=22) and old (*N*=11) mice. Data are from three independent experiments. (**j**) Plasma levels of thrombopoietin in young (*N*=5) and old mice (*N*=5) each measured in triplicate by ELISA. Data are from a single experiment. All data are mean values±s.e.m. *P<0.05; **P<0.01; ***P<0.001 (Student's *t*-test).

The production of mature haematopoietic cells from HSCs proceeds through a hierarchy of increasingly lineage-restricted progenitors^29^. To determine to what degree the increased HSC platelet bias affected progenitor formation, we compared the prevalence of lymphoid^30^,^31^ and myelo-erythroid^25^ progenitor populations between young and old mice. As previously observed^15^,^18^, and consistent with the functional analysis of HSC output, lymphoid-primed multi-potent progenitors were decreased in old mice (Fig. 5a), as was the number of downstream CLPs (Fig. 5b). Analysis of phenotypic myelo-erythroid progenitors showed no change to the total Lin^−^Sca-1^−^c-Kit^+^ population, or to the number of myeloid (preGM, GMP) or erythroid progenitor cells (preCFU-E, colony-forming units (CFU)-E), but a significant increase in MkPs (Fig. 5c–e). Measurement of progenitors in functional assays confirmed the phenotypic progenitor analysis, Mk progenitors (measured as CFU-Mk) were increased (Fig. 5f), whereas myeloid progenitors were unaltered (Fig. 5g), and B-cell progenitors drastically decreased (Fig. 5h). In old mice this increase in Mk progenitors was also accompanied by increased peripheral blood platelets (Fig. 5i), whereas we did not observe higher plasma levels of thrombopoietin, the primary extrinsic regulator of megakaryopoiesis and platelet production (Fig. 5j), in agreement with the increased Mk/platelet-lineage output being intrinsically driven.

### FOG-1 sustains Vwf^+^ HSCs and myeloid HSC bias

**Figure 6:**
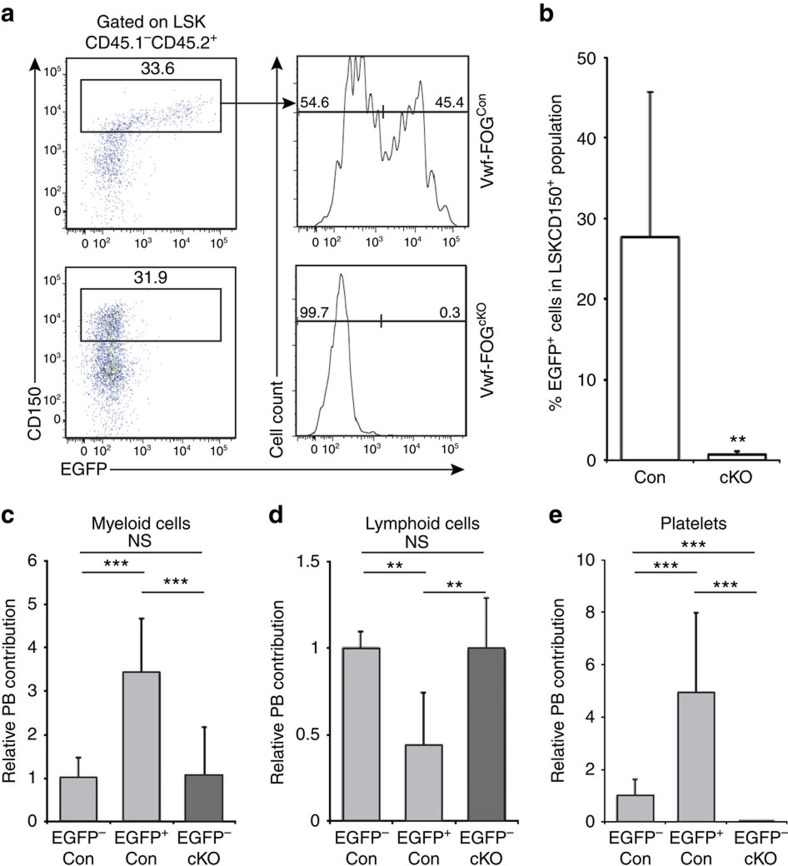
FOG-1 is required for maintenance of myeloid-biased HSCs. (**a**) Representative FACS plots showing CD150 and Vwf-EGFP expression in LSK cells from Vwf-FOG-1^Con^ and Vwf-FOG-1^cKO^ transplanted recipients. Numbers show percentages relative to parental gates. (**b**) Percentage of EGFP^+^ cells in test cell-derived (CD45.1^−^CD45.2^+^) LSKCD150^+^ population from Vwf-FOG^Con^ (Con; *N*=10) and Vwf-FOG^cKO^ (cKO; *N*=8) transplanted mice. **P<0.01 (Student's *t*-test). Data are from two independent experiments. (**c**) Sixteen-week peripheral blood myeloid output from CD45.1^−^CD45.2^+^Lin^−^ Vwf-EGFP^−^ and Vwf-EGFP^+^ fractions isolated from Vwf-FOG-1^Con^ and Vwf-FOG-1^cKO^ primary recipients (FOG1^Con^ EGFP^−^: *N*=13; FOG1^Con^ EGFP^+^: *N*=7; FOG1^cKO^ EGFP^−^: *N*=10). The fraction of CD45.2 myeloid cells was normalized to the overall CD45.2 reconstitution. All data are mean values±s.d., after normalization to the mean value the FOG1^Con^ EGFP^−^ fraction. ***P<0.0001, **P<0.01 (Student's *t*-test). Data are from two independent experiments. (**d**) Sixteen-week peripheral blood lymphoid output analysed and represented as in **c**. (**e**) Sixteen-week peripheral blood platelet output analysed and represented as in **c**, except that significance was measured using the Mann–Whitney *U*-test.

Our results established an anti-correlation between platelet programming of HSCs and lymphoid lineage output. To investigate if platelet-lineage priming could be directly involved in suppression of lymphoid differentiation we ablated *Zfpm1* (encoding FOG-1) in HSCs. To circumvent the lethality associated with pan-haematopoietic *Zfpm1* deletion^24^, we competitively transplanted Vwf-FOG^cKO^ (*Vwf-EGFP*^tg/+^;*Zfpm1*^fl/fl^;*Mx1-Cre*^tg/+^) and Vwf-FOG^Con^ (*Vwf-EGFP*^tg/+^;*Zfpm1*^fl/fl^;*Mx1-Cre*^+/+^) BM cells (CD45.2 allotype) along with CD45.1 wild-type competitor BM cells into irradiated CD45.1/2 recipients. After reconstitution, *Zfpm1* deletion was induced, and 10 weeks post-induction the CD45.2^+^ LSK compartment was analysed. While FOG-1 is not required for HSC engraftment^24^, we observed that Vwf^+^ HSCs were strikingly absent from Vwf-FOG^cKO^ transplanted recipients (Fig. 6a,b), in agreement with the general loss of platelet/erythroid-lineage priming in FOG-1-deficient HSCs (ref. 24). To address if Vwf-FOG^cKO^ HSCs were functionally lymphoid-biased, we compared their long-term lineage readout to Vwf^+^ and Vwf^−^ HSCs from Vwf-FOG^Con^ mice. To avoid assumptions about HSC surface phenotypes, which could be altered by *Zfpm1* inactivation, we transplanted all CD45.1^−^CD45.2^+^Lin^−^ cells (Supplementary Fig. 7), and readout their long-term lineage contributions after 16 weeks, as previously described in ref. 12. As expected^12^, Vwf^+^ Vwf-FOG^Con^ (control) HSCs showed a higher myeloid/platelet output (Fig. 6c,e) than Vwf^−^ Vwf-FOG^Con^ HSCs, which were more lymphoid-biased (Fig. 6d). Vwf-FOG^cKO^ HSCs (which were all Vwf^−^) showed the same lymphoid-biased reconstitution pattern as Vwf^−^ Vwf-FOG^Con^ HSCs, and as previously described^24^, failed to produce platelets (Fig. 6e), compatible with FOG-1 being required to sustain the platelet-primed Vwf^+^ LT-HSC subset, and for the generation of Vwf^+^ myeloid-biased HSCs.

## Discussion

We here identify increased platelet bias as a key feature of haematopoietic ageing in general, and of aged HSCs in particular, manifested by the expansion and predominance of a novel and functionally distinct HSC subtype in the aged haematopoietic system. Using both the *Vwf-EGFP* transgenic reporter and global single-cell transcriptome profiling, we observe a highly significant increase in the number of HSCs expressing high levels of platelet-lineage-specific genes in old, compared with young mice. In fact, gene expression profiling of single Vwf^+^ and Vwf^−^ LT-HSCs showed increased platelet priming of both subsets. This was paralleled by a distinct increase in the proportion of HSCs with functional platelet bias: both Vwf^+^ and Vwf^−^ LT-HSC populations derived from old mice generated a significantly higher proportion of Pl-HSC clones when transplanted at the single-cell level. Downstream of the HSC compartment this was reflected in an increased number of both phenotypically and functionally defined Mk progenitors and, in agreement with previous studies, a higher peripheral blood platelet count^16^,^21^,^32^. In contrast, no increase in myeloid progenitors was observed, whereas lymphoid progenitors were significantly decreased, as previously reported^15^,^18^. Together, these data show that the aged haematopoietic system is platelet-lineage biased, and that functional changes to the HSC compartment can account for this, reflected in increases in both the proportion of platelet-biased HSCs and in their level of intrinsic platelet bias. The increased platelet bias was associated with decreased lymphoid output already at the lymphoid progenitor level. The present and previous data^12^ show that Ly-HSCs derive primarily from the Vwf^−^ LT-HSC population. We observed both a decreased proportion of Vwf^−^ HSCs and a lower frequency of Ly-HSCs within this population in old mice, together leading to a significantly decreased prevalence of Ly-HSCs among aged HSCs, in agreement with previous reports^10^,^33^. These results indicate that age-dependent myeloid bias among leucocytes may primarily be due to decreased lymphopoiesis, rather than increased myelopoiesis, and that this decrease directly correlates with increased platelet priming of HSCs.

While the existence of distinctly lineage-biased HSC subtypes has been abundantly demonstrated little is known about their specification. Extrinsic signals such as Ccl5 (ref. 34) and Wnt5a (ref. 17) have been suggested to promote myeloid bias of HSC lineage output, but intrinsic molecular specifiers of HSC subtypes remain to be identified. We here demonstrate that maintenance of Vwf^+^ HSCs is dependent on FOG-1, a GATA co-factor we previously found to be essential for both commitment to the megakaryocyte lineage and HSC platelet priming^24^. In addition, we observe that FOG-1-deficient HSCs display the lymphoid-biased output normally associated with Vwf^−^ HSCs (ref. 12). This would be consistent with HSC platelet priming suppressing subsequent lymphoid lineage commitment and/or differentiation. The decreased prevalence of lymphoid-bias of aged HSCs could therefore potentially be a consequence of their increased platelet bias. As lymphoid gene expression is barely detectable in HSCs, a possible mechanism is epigenetic suppression of lymphoid genes driven by the platelet-lineage transcriptional programme, as suggested by the increased methylation of the promoters of both B- and T-lineage-specific genes during ageing^35^. Further studies will be needed to address this possibility.

The absolute number of HSCs expands significantly over time, both when measuring phenotypic and functional HSCs^5^,^10^,^13^,^15^. In contrast, the repopulating ability of aged HSCs has been reported to decline, both in terms of frequency of engraftment on transplantation^11^ and level of contribution to the peripheral blood^14^,^15^. Importantly, these results were generated exclusively using leucocyte output to assess HSC engraftment, as measurement of platelet output was precluded in previous transplantation models. We here show that when lineage readout is extended to include platelets (using the *Vwf-EGFP* reporter) there is no decrease in engraftment frequency of aged HSCs, and that in fact Vwf^+^ and Vwf^−^ aged HSCs showed a slightly higher frequency of engraftment compared with their young counterparts, although the difference was not statistically significant. The difference between our results and those previously obtained can therefore be explained by the high proportion of distinct and previously unrecognized platelet-restricted HSCs present in the Vwf^+^ HSC fraction of old mice, as in the majority of engrafted HSCs only platelet output was detected, in contrast to young mice where platelet-restricted HSCs were comparatively rare (1/19 observed). Due to the preferential expansion of Vwf^+^ HSCs in old mice, the Vwf^+^ subset constituted as much as 90% of aged HSCs. Together, these observations indicate that more than half of aged HSCs are represented by these functionally platelet-restricted HSCs, and therefore our ability to detect platelet leads to an increase in the measured repopulation frequency sufficient to account for the previously reported circa 2-fold decrease in engraftment of aged HSCs when relying exclusively on leucocyte output for their detection^11^.

As previously described, we do observe a lower contribution of engrafting aged HSCs to peripheral blood reconstitution^11^,^13^,^15^. Consistent with the engraftment data, this does not appear to be due to a defect in HSC self-renewal, as we observe that young and aged HSCs engraft the HSC compartment with similar efficiencies, as previously observed^14^,^15^. We do, however, observe a decrease in the number of HSCs expressing genes involved in mitotic entry (*Ccna2*, *Ccnb1*, *Cdk1* and *Cdc25*), consistent with the previously observed decrease in cell division of aged HSCs, contributing to a decreased output of differentiated cells. It is also possible that HSC cell divisions become biased towards symmetrical self-renewal division during ageing, which would contribute both to age-dependent HSC expansion and to decreased cellular output of potential relevance, also for the enhanced propensity towards leukaemic transformation with age. This, however, remains to be investigated.

Overall, our results demonstrate that increased molecular and functional platelet bias of HSCs, including a dramatic expansion of a previously unrecognized subset of aged HSCs with platelet-restricted lineage output, is a key feature of haematopoietic ageing, and that this directly contributes to the age-associated imbalance between myeloid and lymphoid leucocyte output. Our finding that FOG-1 is essential for maintenance of the Vwf^+^ platelet-biased HSC subset, identifies for the first time an intrinsic regulator of HSC subtype identity. Finally, the single-cell transcriptome-based identification of gene expression changes associated with HSC ageing provides a resource useful for investigation of the role of other regulators on the aged HSC phenotype.

## Methods

### Mouse strains

*Vwf-EGFP* bacterial artificial chromosome (BAC) transgenic mice (CD45.2) were previously described^12^. Young mice were 2–3 months old, and old mice 20–25 months old. Both male and female mice were used. For generation of Vwf-FOG-1^Con^ or Vwf-FOG-1^cKO^ composite lines, *Vwf-EGFP*^tg/+^ mice were bred with mice carrying a conditional *Zfmp1* allele^36^ as well as the interferon-inducible *Mx1-Cre* transgene^37^. All experimental protocols were approved by the UK Home Office, and the Ethical Review Committee of the Oxford University Medical Sciences Division.

### Poly(I-C) injections

Conditional deletion of *Zfmp1* gene by induction of *Mx1-Cre*-mediated recombination was performed as previously described^38^. Briefly, 5 weeks after competitive transplantation, Vwf-FOG transplanted mice received three injections of 225 μg p(I-C; GE Healthcare, UK) at 2 day intervals.

### Flow cytometry and cell sorting

Purified HSCs (LSKCD150^+^CD48^−^CD34^−^), myelo-erythroid progenitors^25^ and CLPs (ref. 39) were sorted or analysed using either FACS AriaIIu or Fortessa x20 cytometers (BD Biosciences), respectively. Adult BM cells were stained with lineage markers, either c-Kit enriched (AutoMACS, Miltenyi Biotec) or without enrichment were Fc-blocked (except myelo-erythroid progenitors) and stained with anti-mouse^12^ antibodies listed in Supplementary Data 4. A wild-type mouse BM cells were used as negative control for EGFP fluorescence. Data analysis was performed with FlowJo analysis software (TreeStar Inc).

### Blood analysis

Platelet counts were measured in EDTA-blood samples from young and old *Vwf-EGFP* transgenic mice using a Hemavet 950 LV blood analyser (Drew Scientific). Plasma thrombopoietin levels were determined by ELISA in 1:20 diluted samples using the Quantikine M kit (R&D) according to the manufacturer's instructions.

### Competitive transplantation assays

Vwf^−^ (50–75) and 50–75 Vwf^+^ LSKCD150^+^CD48^−^CD34^−^ cells (CD45.2) isolated from young (8–12 weeks) or old (20–25 months) *Vwf-EGFP* transgenic mice were intravenously injected along with young wild-type unfractionated C57Bl/6 BM cells (CD45.1; ratio of 4,000 competitor cells/purified HSC) into lethally irradiated CD45.1 recipients (1,050 cGy, split dose). Lin^−^CD45.1^−^CD45.2^+^Vwf^−^ and Lin^−^CD45.1^−^CD45.2^+^Vwf^+^ cells were sorted from primary recipients transplanted with Vwf-FOG-1^Con^ and Vwf-FOG-1^cKO^ cells at 10 weeks post-poly(I-C) induction. Sorted cells were injected into secondary lethally irradiated CD45.1/2 recipients, at cell numbers proportional to their relative frequencies in the primary recipients, along with 2.5 × 10^5^ wild-type unfractionated BM cells (CD45.1). Peripheral blood leucocyte and platelet reconstitution analysis were performed at 16 weeks post-transplantation to determine *in vivo* lineage output of FOG-1-deficient Vwf^+^ and Vwf^−^ HSCs.

### Single cell and limiting dilution transplantations

For single-cell transplantations, single Vwf^+^ or 5 Vwf^−^ HSCs (CD45.2 allotype) were FACS-sorted using the FACSAriaII automated cell deposition unit, and collected into individual wells of a round-bottomed 96-well plate containing 100 μl of transplantation media: Iscove's modified Dulbecco's medium (IMDM) containing 1% penicillin–streptomycin, 1% β-mercaptoethanol, 1% L-glutamine and 20% BIT 9,500 serum substitute (Stem Cell Technologies). To ensure that single cells were deposited, single fluorescent beads were sorted, and after microscopic inspection >98% of the wells were found to contain a single bead, and no wells more than one bead. After sorting single cells, 1 million *W*^*41*^*/W*^*41*^ (c-Kit-deficient; CD45.1 allotype) unfractionated competitor BM cells in 100 μl volume were added to each well. Cells were mixed, loaded into a Terumo Myjector syringe and injected into the lateral vein of lethally irradiated (1,050 cGy, split dose, 4 h apart) recipients (CD45.1 allotype). Peripheral blood cells from recipient mice were analysed at 6, 10 and 16 weeks post-transplantations.

### Generation of FOG-1 BM chimeras

5 × 10^5^ unfractionated BM (CD45.2) cells from Vwf-FOG-1^Con^ (*Vwf-EGFP*^tg/+^; *Zfpm*^fl/fl^; *Mx1-Cre*^+/+^) and Vwf-FOG-1^cKO^ (*Vwf-EGFP*^tg/+^; *Zfpm*^fl/fl^; *Mx1-Cre*^tg/+^) mice were intravenously injected along with 5 × 10^5^ unfractionated BM competitor cells (CD45.1) into lethally irradiated CD45.1/2 recipients (1,070 cGy, split dose). Donor chimerism in peripheral blood was assessed 5 weeks after transplantation. Subsequently, *Zfpm1* deletion was induced by p(I-C) injections and donor contribution to platelets and leucocytes was determined 4 weeks after first injection to determine recombination efficiency.

### Cell culture

Colony-formation assays were performed as follows. For CFU-Mk measurement, 100.000 unfractionated BM cells from young and old *Vwf-EGFP* mice were plated per slide in quadruplicate in Mega-Cult-C media (Stem Cell Technologies, Vancouver, Canada) supplemented with 50 ng ml^−1^ human thrombopoietin, 10 ng ml^−1^ murine interleukin-3, 20 ng ml^−1^ human interleukin 6 and 50 ng ml^−1^ human interleukin 11 (Peprotech). Cultures were acetone-fixed and acethylcolinesterase (AchE) stain was performed as per manufacturer's instructions. AchE^+^ colonies were scored at day 11. For CFU-GM measurement, 50.000 unfractionated BM cells from young and old *Vwf-EGFP* mice were plated in M3534 media in triplicate and colonies scored at day 8. For CFU-B measurement, 200.000 unfractionated BM cells from young and old *Vwf-EGFP* mice were plated in triplicate in M3630 media and colonies scored at day 8. All media for CFU assays were from Stem Cell Technologies (Vancouver, Canada).

### Gene expression analysis

For single-cell gene expression analysis, complementary DNA (cDNA) synthesis and specific target gene amplification were done using the CellsDirectOne-StepqRT–PCR kit (Invitrogen). Single cells were directly sorted into 96-well PCR plates, each well with 10 μl Cells Direct One-Step qRT–PCR mix containing 1 × Cells Direct Reaction Mix, 1.2 ml RT-Taq mix, 0.1 ml SUPERase-In RNase Inhibitor (Ambion) and 200 nM primers (Supplementary data 5). The cDNA was pre-amplified (22 cycles) and pre-amplified samples were then diluted to 50 μl with Tris-EDTA (TE) buffer. Each sample of 2.5 μl was used for multiplex quantitative PCR using BioMark 48.48 Dynamic Array platform (Fluidigm) and TaqMan Gene Expression Assays (Applied Biosystems; Supplementary Data 5). Data were analysed using BioMark Real-Time PCR Analysis Software. For normalization, ΔCt values were calculated relative to *Kit* expression in each cell and single cells without *Kit* expression were excluded from data. 111 young Vwf^+^ LT-HSC, 163 old Vwf^+^ LT-HSC, 123 young and 167 old Vwf^−^ LT-HSCs were included in the final analysis.

### Single-cell RNA sequencing library construction

Lin^−^Sca-1^+^c-Kit^+^CD150^+^CD48^−^ cells (2,000) from young and old *Vwf-EGFP* mice were sorted in 2 μl filter-sterilized PBS with 50% FCS (ref. 12). Single cells were captured on a small-sized (5–10 μm cell diameter) C1 Single-Cell Auto Prep IFC for messenger RNA Sequencing (Fluidigm) using the Fluidigm C1 system. Cells were loaded onto the chip at a concentration of ∼400 cells μl^−1^ and imaged by phase-contrast microscopy to check single cell per capture site. Sixty-one young HSCs and 74-old HSCs were captured and used for RNA-seq. Cells were lysed and whole-transcriptome amplified cDNA prepared on the C1 Fluidigm chip according to the manufacturer's protocol, using SMARTer Ultra Low RNA kit for Illumina (Clontech). Single-cell cDNA libraries were quantitated by an Agilent Bioanalyzer using High-Sensitivity DNA chip. Illumina libraries were constructed in 96-well plates using the Illumina Nextera XT DNA Sample Preparation kit according to a protocol supplied by Fluidigm. Indexed libraries size and quality was checked using Agilent High-Sensitivity DNA chip. The concentration of indexed libraries was determined using Qubit High-Sensitivity DNA kit (Invitrogen). Libraries were pooled to a final concentration of 4–5 nM and were sequenced on an Illumina HiSeq 2,000 (single end 51 base pair reads) at SciLifeLab, Karolinska Institutet, Stockholm, Sweden.

### Single-cell RNA sequencing analysis

Short reads (51 bp) from 135 HSCs were mapped using Tophat (version 2.0.10 (ref. 40)) against the mouse genome (mm10 assembly) with a supplied set of RefSeq gene model annotations as the input. The mapping parameters ‘-g 1' was used to allow one alignment to the reference for a given read. Supplementary Data 6 shows the number of mapped reads, percentage of mapping and the number of detected genes per single cell. Cells with <500.000 mapped reads with per cent of mapping to the mitochondrial chromosome>10% and<3,000 detected genes were excluded from further analysis^20^. 52 young and 62-old HSCs (in total 114 out of 135 or 84% of cells) fulfilled these criteria. Expression values were quantified based on RefSeq using rpkmforgenes^41^. CPM reads (CPM) value was then calculated using the ‘cpm' function in edger (ref. 42). Genes (11,910) were selected for PCA analysis based on coefficient of variation (CV≥1) and mean of expression values across all cells (log2(CPM)>0). The 100 genes with the highest absolute correlation coefficient (PCA component loadings, one of the first three components) were used for hierarchical clustering analysis (distance='pearson correlation'; method='complete'). R scripts were used to perform hierarchical clustering, and to visualize on a log2 of CPM scale. Differentially expressed genes analysis was performed using nonparametric Wilcoxon test for the expression level and Fisher's exact test for the expressing cell frequency. *P* values generated from both tests were then combined using Fisher's method and were adjusted using Benjamini–Hochberg. GSEA was performed using GSEA software (http://www.broadinstitute.org/gsea) with 1,000 permutations of the gene sets and log2 ratio of classes as metric for ranking genes. Gene sets used in this study have been previously published^24^,^25^. Pathways enrichment analysis was performed using the Pathway tool of MetaCore from Thomson Reuters (Version 6.15).

### Statistical analysis

Data are presented as mean±s.e.m. or±s.d. values, as indicated. Two-tailed Student's *t*-test was used to determine statistical significance of gene expression levels, except for global single transcriptome analysis, where Wilcoxon's and *χ*^2^ tests were combined using Fischer's method. Chi-square test was used for expressing cell frequency analysis. For regular sample sizes, we set statistical significance at *P*<0.05. For large sample sizes (>100) this was set at *P*<0.005. The Kolmogorov–Smirnov test was used to analyse co-expression of lineage-specific genes. Two-tailed Student's *t*-test (for population transplants) and Mann–Whitney *U*-test (for single-cell transplants) were used for comparison of reconstitution levels, and Fisher's exact test for analysis of the frequency of biased HSC subtypes. Sample sizes were based on previous experience. No data were excluded. Analysis was not blinded or randomized.

## Additional information

**Accession codes:** The single-cell RNA sequencing data has been deposited in the Gene Expression Omnibus under accession code GSE70657.

**How to cite this article:** Grover, A. *et al*. Single-cell RNA sequencing reveals molecular and functional platelet bias of aged haematopoietic stem cells. *Nat. Commun.* 7:11075 doi: 10.1038/ncomms11075 (2016).

## Supplementary Material

Supplementary Information Supplementary Figures 1-7

Supplementary Data 1 Pathways enriched in young compared to old HSCs. The top 20 Metacore pathways enriched in genes up-regulated in young, compared to old, LSKCD150+ CD48− HSCs. The pathway name and P-value are shown.

Supplementary Data 2 Pathways enriched in old compared to young HSCs. The top 20 Metacore pathways enriched in genes up-regulated in old, compared to young, LSKCD150+ CD48− HSCs. The pathway name and P-value are shown.

Supplementary Data 3 Gene expression levels and frequencies in young and old HSCs. The top 60 genes up-regulated in old, compared to young, LSKCD150+ CD48− HSCs. The gene ID, mean of CPM values (log2), fold regulation, and adjusted P-value are shown.

Supplementary Data 4 Antibodies used for flow cytometry. The conjugate, clone used, supplier and usage is shown for antibodies used in flow cytometry.

Supplementary Data 5 TaqMan assays used for gene expression analysis. The gene identifiers and assay number for TaqMan assays used for single cell gene expression analysis are shown.

Supplementary Data 6 Mapping of single HSC transcriptomes. For each cell the number of input reads, mapped reads, percentage of mapping, and the number of detected genes at >= 1 RPKM per single cell are shown. For QC purposes, the amount and percentage of reads mapped to the mitochondrial chromosome, and the number of genes detected at log2(CPM+1)>2 are shown. It is indicated which cells pass the QC criteria described in the Methods section.
