# Supplementary material for: Single-cell RNA sequencing reveals molecular and functional platelet bias of aged haematopoietic stem cells
Source: Nat Commun. 2016 Mar 24;7:11075. doi: 10.1038/ncomms11075 (PMC4820843; doi:10.1038/ncomms11075)
Supplement: Supplementary Information — Supplementary Figures 1-7 [file ncomms11075-s1.pdf]

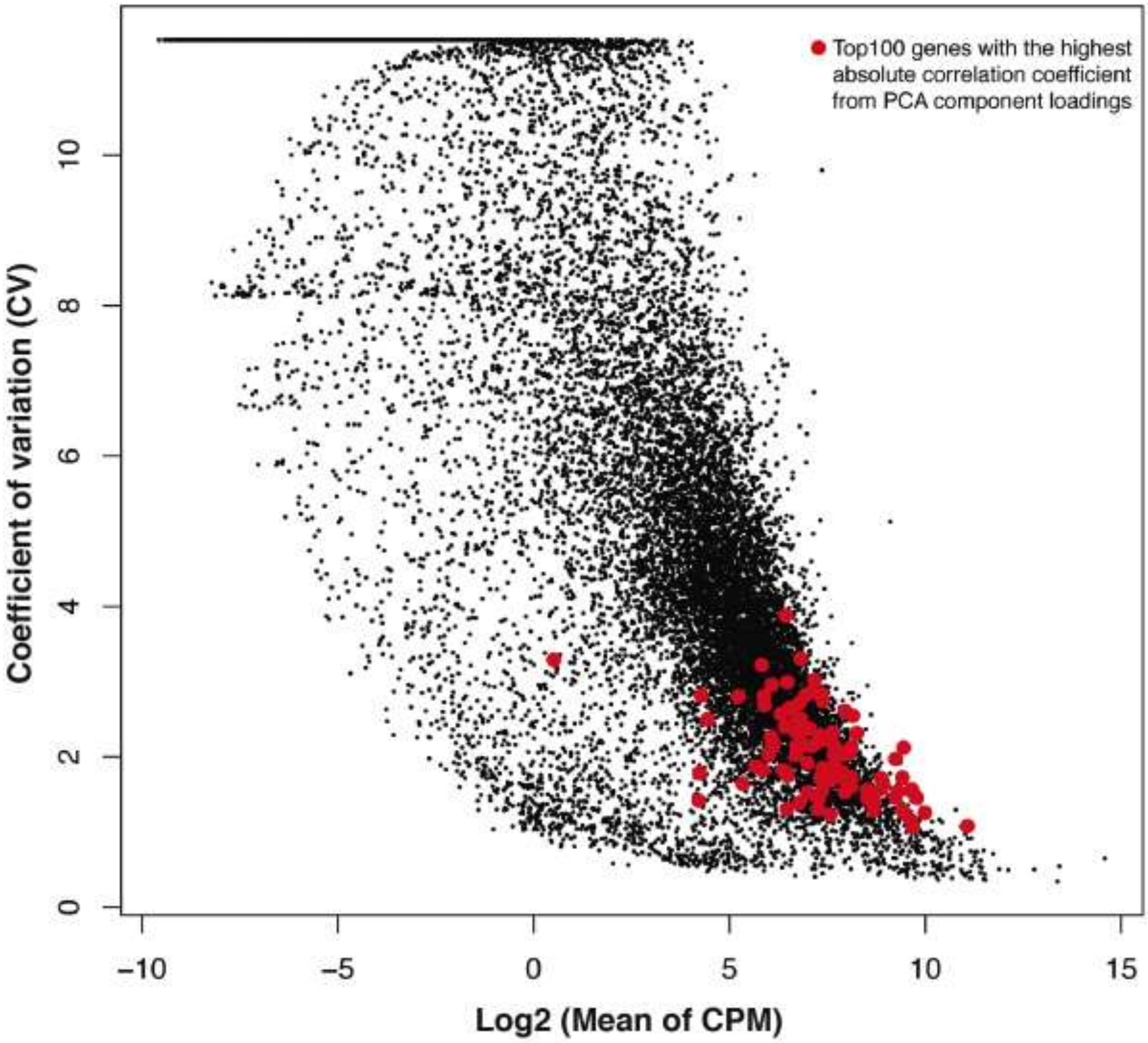

Supplementary Fig. 1: Expression level and variance of genes used for HSC clustering.

Scatter plot of CV versus mean expression for all detected genes over all cells in the dataset. Each dot represents a single gene. The 100 genes used for clustering are highlighted in red.

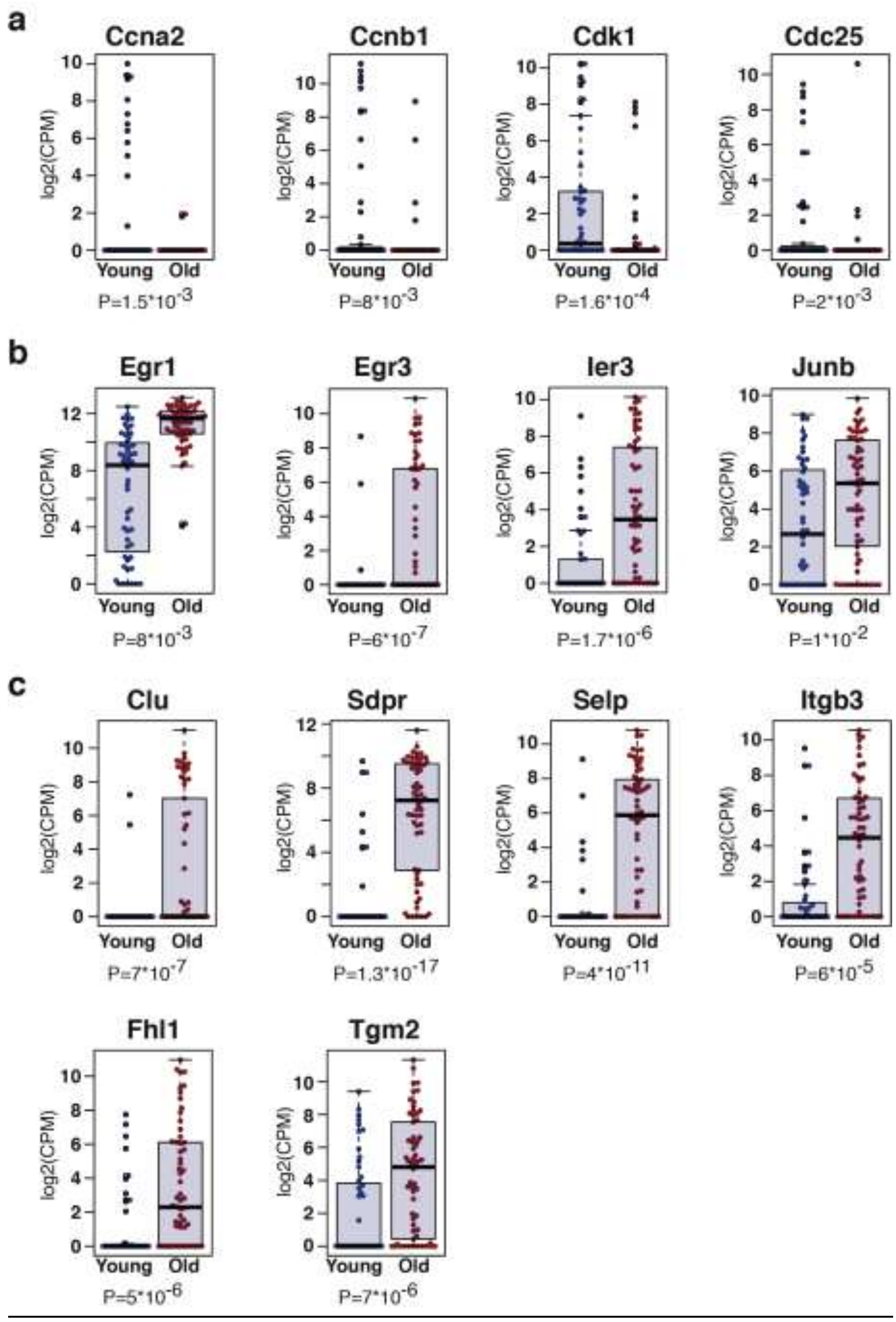

Supplementary Fig. 2: Gene expression in single HSCs.

a) Expression of key genes associated with mitosis program in single LSKCD150+CD48– LT-HSCs from Fig. 1a. Each circle represents a single HSC. A total of 52 young and 62 old LT-HSCs were profiled in two separate experiments. P-values for the difference in frequency with which each gene is expressed are shown (Fisher's exact test).

b) Expression of selected immediate-early genes analyzed as in (a).

c) Expression of selected platelet-lineage genes analyzed as in (a).

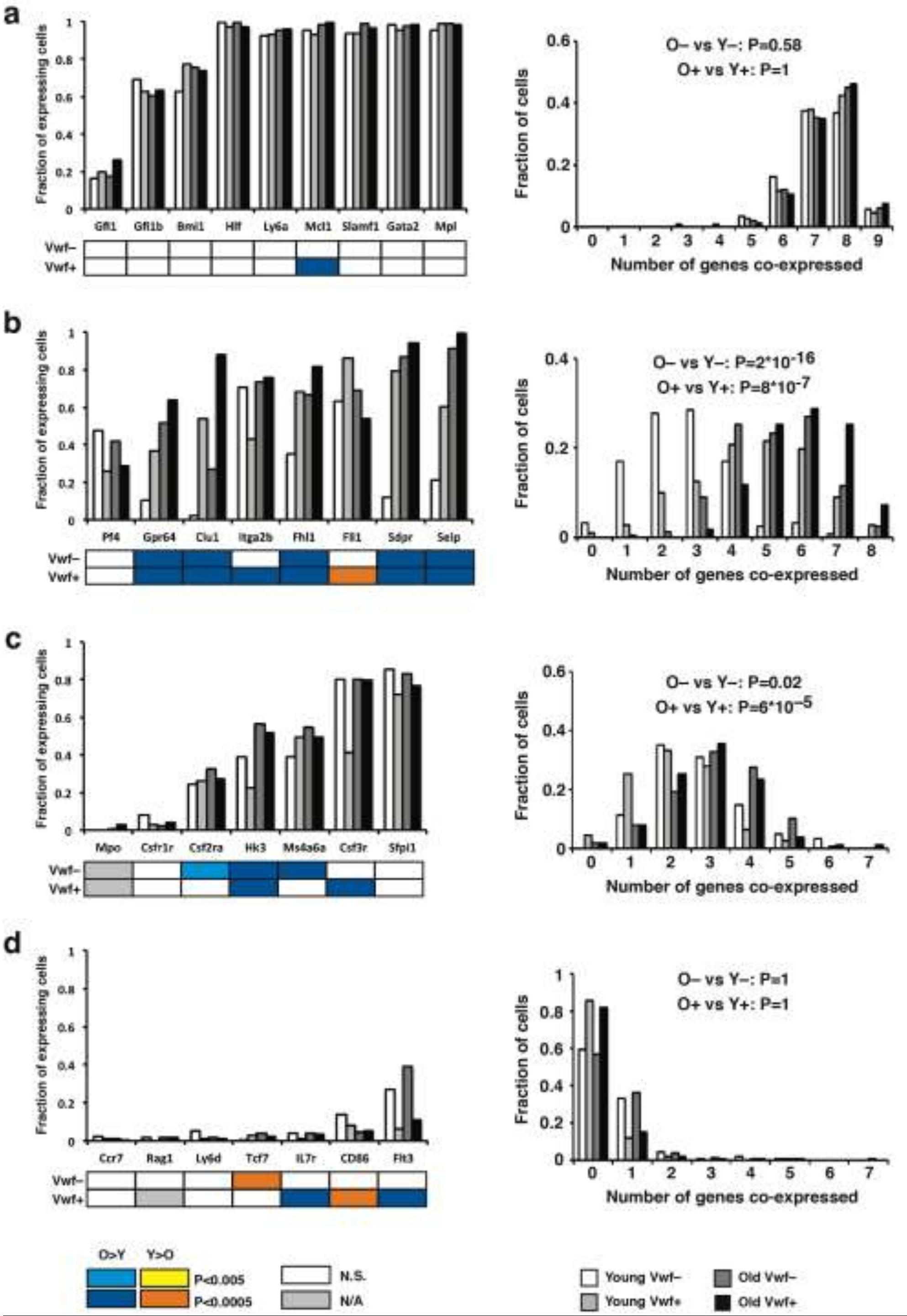

Supplementary Fig. 3: Stem cell and lineage associated gene expression in young and aged LT-HSCs.

a) Expression frequencies of individual genes within the HSC program in single Vwf^{+} and Vwf^{-} LT-HSCs, from young and old mice from **Fig. 2a**. The left histogram shows the frequency with which expression was detected for individual HSC-associated genes. The significance of any difference in expression frequency is indicated by the colored bars below (Chi-square test). The

right histogram shows the frequency of cells expressing a given number of HSC-associated genes, with the significance between young and aged populations determined using the Kolmogorov-Smirnov test. N.S.: not significant; N/A: not applicable (due to lack of expressing cells in one condition).

b) Analysis as in (a) of individual platelet lineage genes.

c) Analysis as in (a) of individual myeloid lineage genes.

d) Analysis as in (a) of individual lymphoid lineage genes.

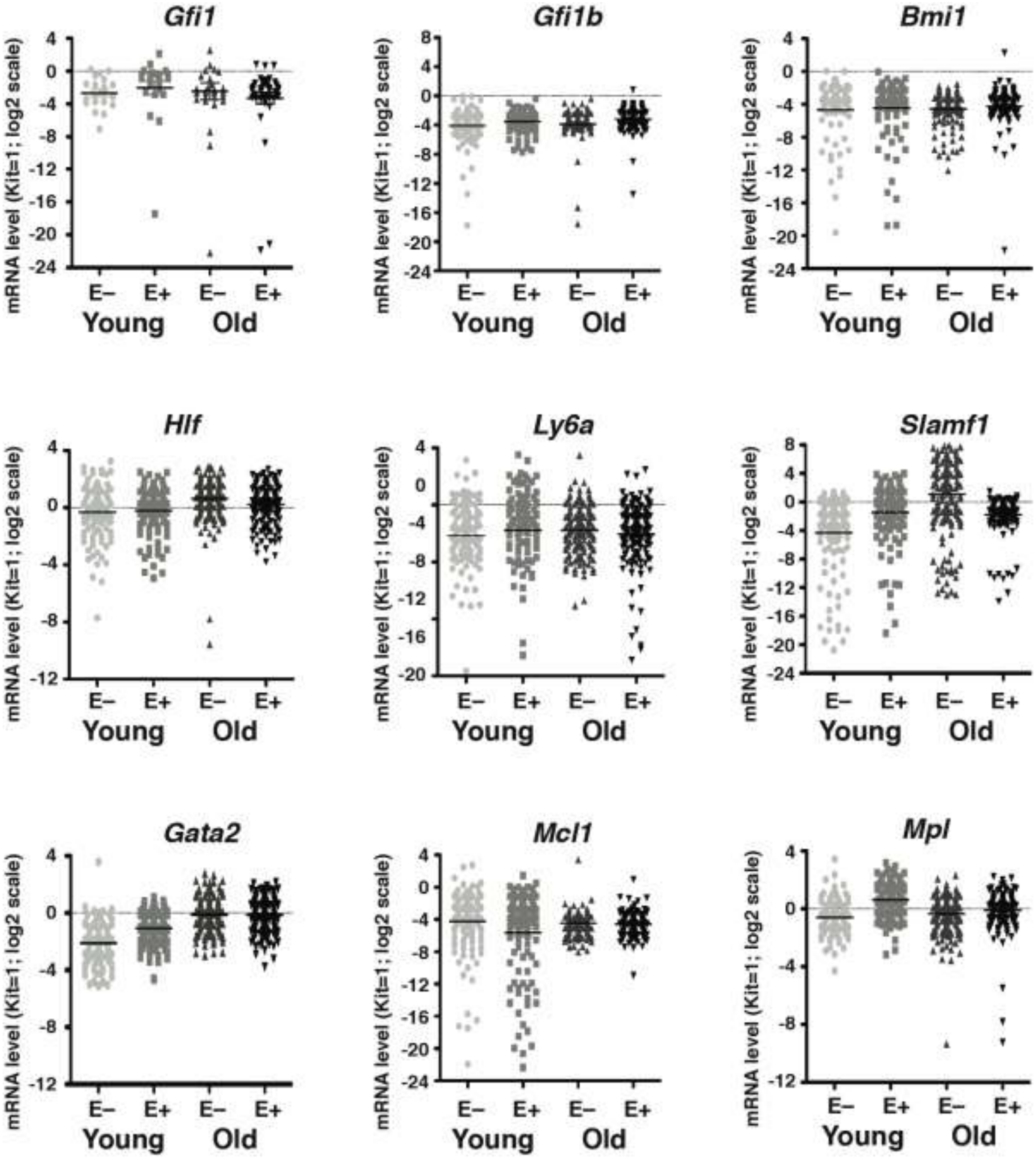

Supplementary Fig. 4: HSC gene expression levels in single LT-HSCs.

Expression levels of HSC-associated genes in single Vwf^{+} and Vwf^{-} LT-HSCs from young and old mice from **Fig. 2a**. Each data point represents the mRNA level in a single cell, normalized to *Kit* expression. Bars show the mean expression value and its standard deviation.

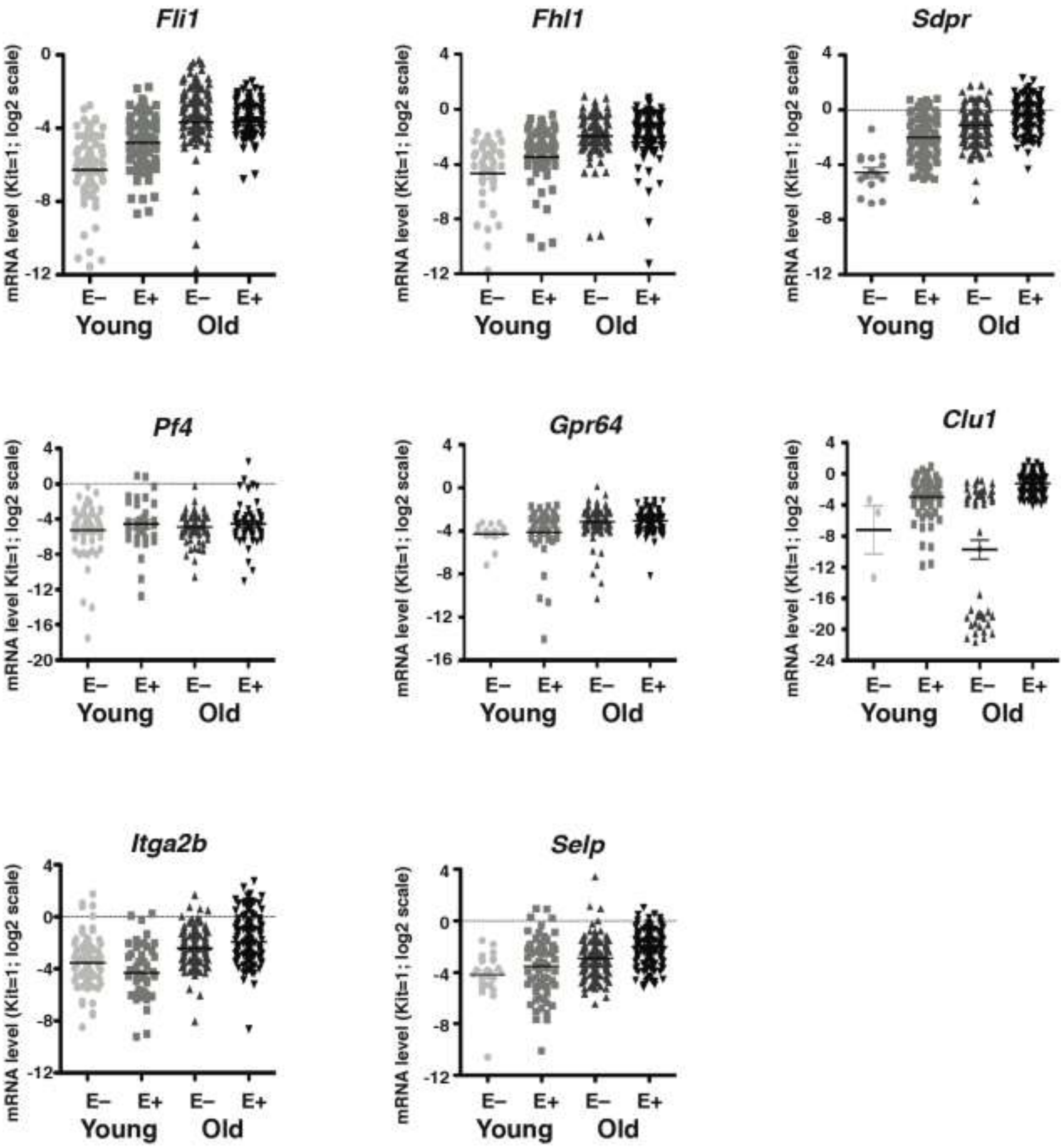

Supplementary Fig. 5: Platelet gene expression levels in single LT-HSCs.

Expression levels of the indicated platelet lineage genes in single Vwf^{+} and Vwf^{-} LT-HSCs, from young and old mice from **Fig. 2a**, presented as in Supplementary Fig. 3.

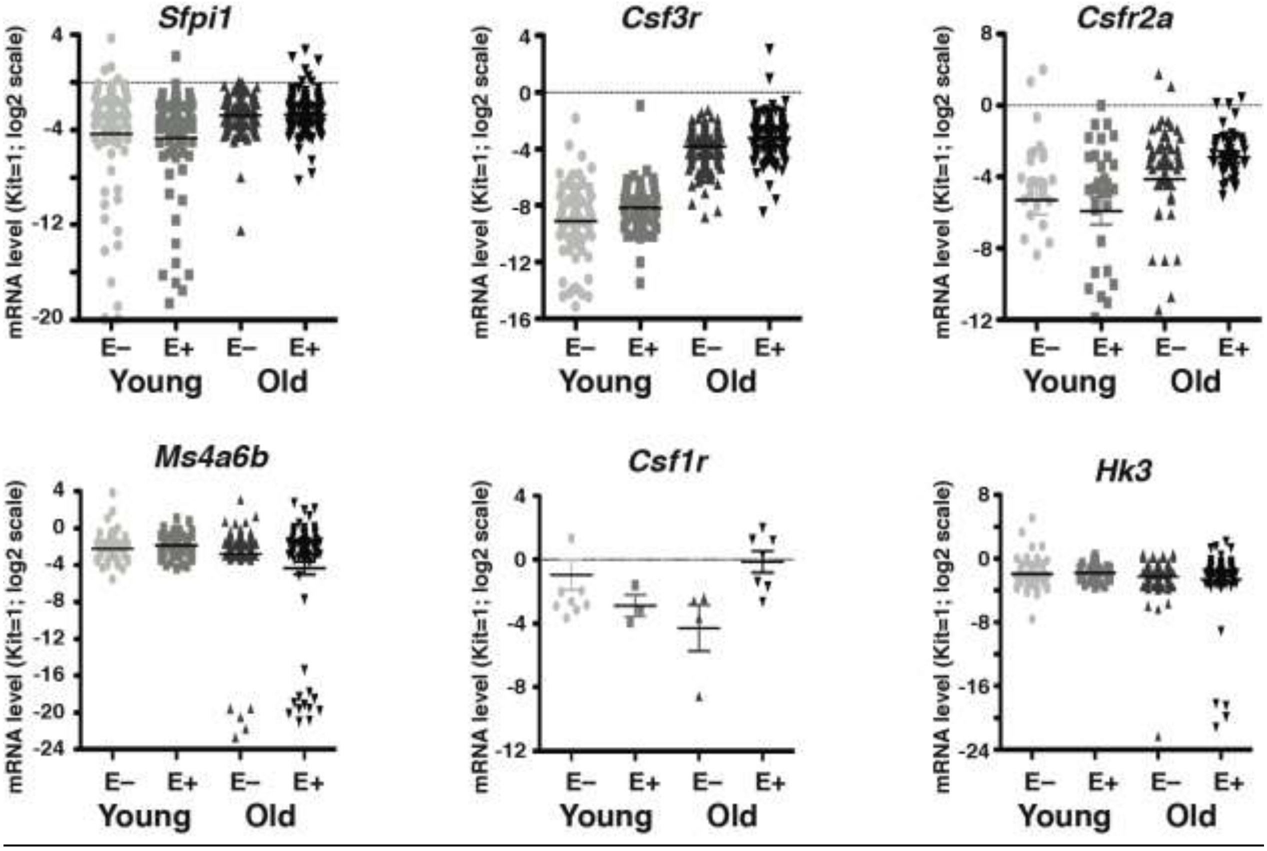

Supplementary Fig. 6: Myeloid gene expression levels in single LT-HSCs.

Expression levels of the indicated myeloid lineage genes in single Vwf^{+} and Vwf^{-} LT-HSCs, from young and old mice from **Fig. 2a**, presented as in Supplementary Fig. 3.

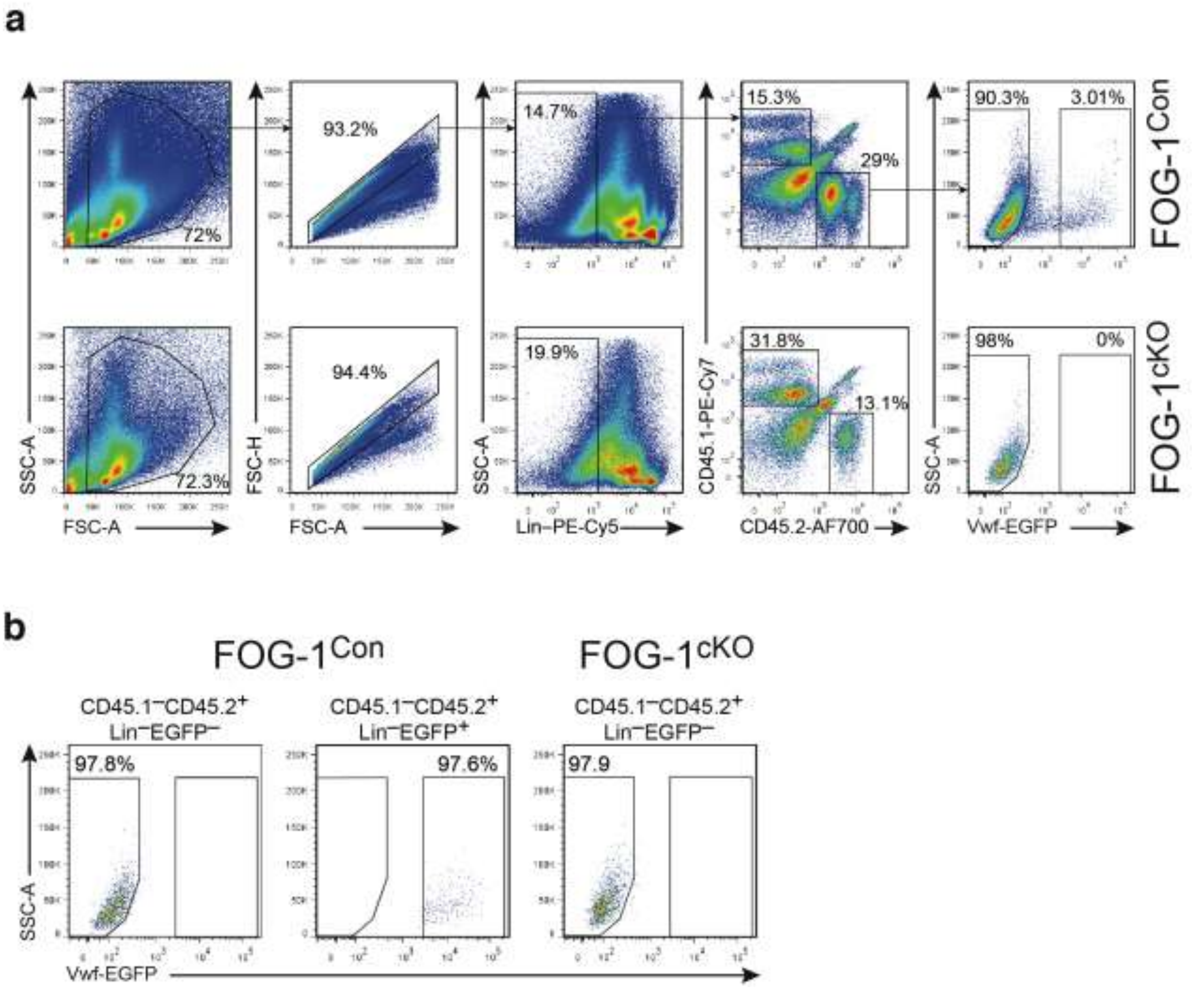

<u>Supplementary Fig. 7: Isolation of CD45.1^{-}CD45.2^{+}Lin^{-}Vwf^{-} and Vwf^{+} cells from Vwf-FOG-1^{Con} and Vwf-FOG-1^{cKO} transplanted mice.</u>

a) As indicated CD45.1^{-}CD45.2^{+}Lin^{-}Vwf^{-} and Vwf^{+} cells were sorted from Vwf-FOG-1^{Con} and CD45.1^{-}CD45.2^{+}Lin^{-}Vwf^{-} cells from Vwf-FOG-1^{cKO} transplanted mice.

b) Each isolated fraction was re-analyzed for sort purity before being competitively transplanted into secondary recipients using cell doses proportional to their abundance in the primary recipients (i.e. maintaining the ratio of Vwf^{+} and Vwf^{-} cells). Data are from 2 independent experiments.
